# Molecular Pathogenesis and Targeted Treatment of Richter Transformation

**DOI:** 10.3390/biomedicines14020347

**Published:** 2026-02-02

**Authors:** Nawar Maher, Amir Karami, Bassam Francis Matti, Alaa Fadhil Alwan, Sayed Masoud Sayedi, Riccardo Moia, Gianluca Gaidano, Samir Mouhssine

**Affiliations:** 1 Division of Hematology, Department of Translational Medicine, Università del Piemonte Orientale and Azienda Ospedaliero-Universitaria di Alessandria, 56121 Alessandria, Italy; nawar.maher@uniupo.it; 2 Division of Hematology, Department of Translational Medicine, Università del Piemonte Orientale and Azienda Ospedaliero-Universitaria Maggiore della Carità, Via Solaroli 17, 28100 Novara, Italy; 20062237@studenti.uniupo.it (A.K.); sayedmasoud.sayedi@uniupo.it (S.M.S.); riccardo.moia@uniupo.it (R.M.); samir.mouhssine@uniupo.it (S.M.); 3 Department of Hematology and Bone Marrow Transplant, Hematology and Bone Marrow Transplant Center, Medical City, Baghdad 12114, Iraq; bassam_francis@iqsoh.org; 4 Department of Clinical Hematology, The National Center of Hematology, Mustansiriyah University, Baghdad 10052, Iraq; ala_sh73@yahoo.com

**Keywords:** chronic lymphocytic leukemia, Richter transformation, molecular pathogenesis, tumor microenvironment, targeted therapy

## Abstract

Richter transformation (RT) represents a rare but highly lethal evolution of chronic lymphocytic leukemia/small lymphocytic lymphoma (CLL/SLL), most frequently manifesting as diffuse large B-cell lymphoma (DLBCL). Despite therapeutic advances in CLL, DLBCL-RT remains characterized by rapid progression, profound treatment refractoriness, and short survival with conventional chemoimmunotherapy, underscoring the need for a refined biological and therapeutic framework. A defining feature of RT is clonal relatedness: most cases arise through linear or branched evolution of the antecedent CLL clone and carry an inferior prognosis compared with clonally unrelated cases that resemble de novo DLBCL. Recent multi-omic data further indicate that clonally related RT commonly originates from minute, transformation-primed subclones detectable years before clinical emergence, shifting RT from a late stochastic event to an early-established evolutionary trajectory. At transformation, recurrent genetic lesions of *TP53*, *CDKN2A*/*B*, *NOTCH1*, and *MYC* cooperate with B-cell receptor-associated programs, epigenetic reconfiguration, and metabolic rewiring toward OXPHOS- and mTOR-driven states, collectively promoting genomic instability and aggressive growth. In parallel, RT develops within a profoundly immunosuppressive microenvironment marked by PD-1-expressing malignant B cells, PD-L1-rich myeloid niches, exhausted T cells, expanded regulatory T cells, and M2-skewed macrophages interconnected by redundant checkpoint and cytokine networks. Therapeutic strategies are rapidly evolving, including pathway inhibitors, immune checkpoint blockade, T-cell-engaging bispecific antibodies, CAR-T therapies, and antibody–drug conjugates. This review integrates current insights into RT pathogenesis, immune escape, and emerging therapies, highlighting opportunities for biomarker-driven patient stratification, rational combinations, and earlier interception of transformation-prone disease.

## 1. Introduction

Richter transformation (RT) is a rare and highly aggressive evolution of chronic lymphocytic leukemia/small lymphocytic lymphoma (CLL/SLL), defined by the development of a high-grade lymphoma, most commonly diffuse large B-cell lymphoma (DLBCL) [1,2,3]. RT occurs in approximately 2–10% of CLL patients over the disease course and represents one of the leading causes of CLL-related mortality [4]. While less frequent subtypes such as Hodgkin lymphoma-RT exist, DLBCL-RT accounts for nearly 90% of cases and is characterized by rapid progression, marked therapy resistance, and historically dismal outcomes, with median overall survival (OS) measured in months with conventional chemoimmunotherapy (CIT) [5].

A central biological distinction in RT is its clonal relationship with the antecedent CLL. Most DLBCL-RT cases are clonally related and arise through linear or branched evolution of the original CLL clone, accumulating high-risk molecular lesions and displaying inferior prognosis compared with clonally unrelated cases, which resemble de novo DLBCL [6]. The introduction of targeted agents in CLL, particularly B cell receptor (BCR) and BCL2 inhibitors, has profoundly altered disease biology, clonal selection, and the landscape of RT, underscoring the need to reinterpret RT pathogenesis in the era of targeted therapy [7,8].

RT is driven by the integration of clonal evolution, genetic and epigenetic alterations, early tumor seeding, metabolic reprogramming, and a profoundly dysregulated immune microenvironment [9]. These mechanisms not only fuel aggressive transformation but also shape immune escape and treatment refractoriness.

Therapeutically, DLBCL-type RT has been historically managed with anthracycline-based CIT regimens extrapolated from de novo DLBCL, most commonly R-CHOP or intensified variants [10]. However, response rates remain low, complete remissions are infrequent, and survival outcomes are poor, with median OS generally limited to less than one year [11,12]. Intensified CIT approaches and alternative regimens have failed to produce durable benefit and are often accompanied by substantial toxicity, while curative strategies such as allogeneic stem cell transplantation are feasible in only a minority of patients [13]. Collectively, these limitations highlight the inadequacy of current standard therapies and underscore the urgent need for more effective, biologically informed treatment strategies. In this context, the therapeutic armamentarium for RT is rapidly expanding, including pathway inhibitors, immune checkpoint blockade, T-cell-engaging strategies such as bispecific antibodies (bsAbs) and chimeric antigen receptor (CAR)-T cells, and antibody-drug conjugates (ADCs) [14]. This review provides an integrated overview of DLBCL-RT, linking molecular pathogenesis, immune microenvironment, and emerging targeted therapies within a unified translational framework.

## 2. Molecular Mechanisms in CLL Transformation to RT

The biological underpinnings of RT reflect a multifaceted process driven by diverse molecular events that progressively reshape the behavior of the underlying CLL clone [9,15]. Studies integrating genomic, epigenomic, transcriptomic, and metabolic data reveal distinct pathways through which CLL can acquire the hallmarks of aggressive lymphoma. These mechanisms encompass changes in clonal architecture, early dissemination of transformation-prone cells, and widespread reprogramming of genetic and non-genetic regulatory networks. The following sections outline the principal molecular processes currently implicated in this evolution.

### 2.1. Clonal Evolution and Lineage Relationship of DLBCL-RT

Clonal relationship represents a central determinant in the biology and clinical behavior of DLBCL-RT [10,16]. In approximately 80% of DLBCL-RT cases, the transformed lymphoma and the antecedent CLL share an identical immunoglobulin heavy chain variable region (IGHV) rearrangement, indicating direct evolution of the pre-existing CLL clone [17]. In contrast, about 20% of cases harbor a completely distinct IGHV configuration, signifying a clonally unrelated de novo DLBCL that arises in the immunologically permissive environment created by the underlying CLL. This distinction carries important prognostic implications: clonally unrelated RT generally exhibits outcomes comparable to de novo DLBCL in patients without CLL, whereas clonally related RT is associated with significantly inferior survival [18].

The biological basis of clonally unrelated RT has only recently been clarified. Ultra-deep next-generation sequencing has demonstrated that, even with sensitivity down to 10^−6^, the DLBCL clone is undetectable during the CLL phase, confirming that these tumors arise as true secondary lymphoid malignancies rather than from an occult CLL subclone [6]. Genomic profiling further shows that clonally unrelated RT is enriched for lesions affecting DNA damage response pathways (e.g., *TP53*, *ATM*) and genes involved in cell-cycle regulation and proliferation (e.g., *NOTCH1*, *ID3*, *MYC*) [18]. Notably, recurrently mutated genes typical of de novo DLBCL, such as *KMT2D*, *CREBBP*, *EP300*, *TNFAIP3*, and those involved in B-cell receptor signaling, are largely absent. Thus, despite their distinct cellular origins, clonally related and clonally unrelated RT converge on a partially overlapping mutational spectrum, likely reflecting shared selective pressures imposed by profound immune dysregulation, microenvironmental abnormalities in CLL lymph nodes, and prior exposure to cytotoxic or targeted therapies [19].

These molecular insights have direct clinical relevance. First, the presence of common genetic vulnerabilities across both RT subtypes suggests the possibility of shared targeted therapeutic strategies. Second, the high prevalence of *TP53* disruption in both forms of RT underscores the importance of assessing *TP53* status in the RT biopsy, given its strong association with chemoresistance across CLL and DLBCL [19]. Finally, because treatment pathways diverge, particularly regarding suitability for hematopoietic stem-cell transplantation, determining clonal relatedness remains essential [20]. Although paired CLL and RT tissue samples are not always available, immunophenotypic surrogates such as PD-1 expression have demonstrated approximately 90% concordance with molecular assessment of clonal relatedness and may assist clinical decision-making when genetic comparison is not feasible [21].

### 2.2. Early Seeding of Clonally Related DLBCL-RT

Recent multi-omic analyses have also reshaped our understanding of the temporal dynamics that underlie clonally related DLBCL-RT. A comprehensive study integrating whole-genome sequencing, epigenomic profiling, and single-cell DNA/RNA sequencing of 19 patients demonstrated that RT frequently originates from minute, pre-existing CLL subclones that are already detectable many years before clinical transformation [6]. These dormant subclones, which could remain at a cancer cell fraction below 1% for up to 19 years, harbor early driver lesions such as *TP53*, *CDKN2A*/*B*, *NOTCH1*, *SF3B1*, and chromatin-modifying gene alterations, and progressively accumulate additional mutations, complex structural variants, and transcriptomic features characteristic of RT prior to overt expansion [6]. Single-cell analyses revealed that divergent *TP53*-mutated subclones can coexist for years, with one eventually gaining a selective advantage and giving rise to RT. Moreover, the study uncovered new mutational processes, including the novel single-base substitution-RT signature linked to prior exposure to alkylating agents, as well as early immunogenetic diversification, with RT-defining IGHV/V(D)J mutations detectable long before transformation. Collectively, the findings provide compelling evidence that clonally related DLBCL-RT is not a late stochastic event but rather the outgrowth of deeply rooted evolutionary lineages established early in CLL pathogenesis, highlighting the need for ultra-sensitive detection strategies and early molecular risk stratification.

### 2.3. Driver Mutations and Genomic Instability

**Figure 1:**
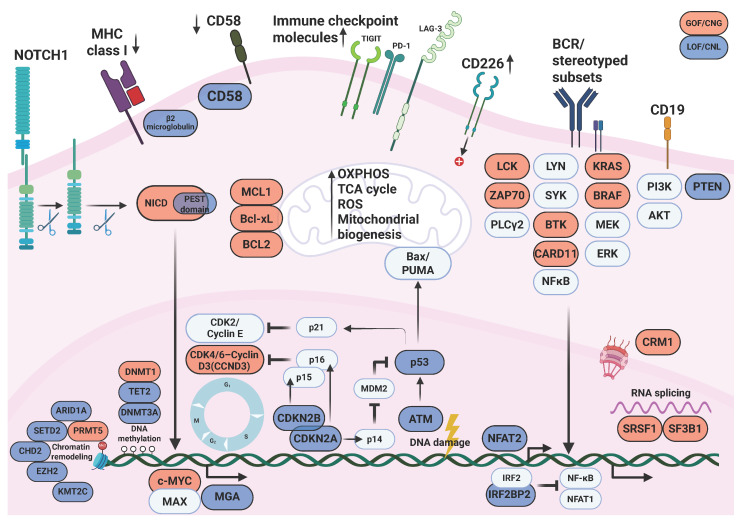
Multi-level molecular mechanisms driving RT. The figure summarizes the molecular mechanism of RT by grouping events at the cell surface, in the cytoplasm/organelles, and in the nucleus. At the receptor and immune-interface level, RT cells can evade immune surveillance by reducing antigen presentation (MHC-I downregulation), weakening immune synapse engagement (CD58 downregulation), and increasing inhibitory checkpoint molecules (PD-1, TIGIT, LAG-3). Dysregulated TIGIT-CD226 signaling, stereotyped BCR/CD19 activation, and sustained NOTCH1 signaling converge to hyperactivate cytoplasmic pathways, including NF-κB, MAPK/ERK, and PI3K-AKT, which are often further amplified by *PTEN* loss and LCK/ZAP70-mediated signal boosting; in parallel, mitochondria increase energy production (OXPHOS/TCA) and biogenesis, while anti-apoptotic BCL2-family dominance neutralizes BH3-only stress signals and prevents BAX/BAK-mediated mitochondrial outer membrane permeabilization. In the nucleus, transformation is sustained by failed DNA-damage/checkpoint control (ATM-p53 disruption, MDM2 p53 suppression, and loss of *CDKN2A*/*B*), alongside epigenetic/chromatin remodeling changes, MYC/MAX hyperactivity with loss of restraining factors such as MGA, and aberrant RNA processing and nuclear export through SF3B1, SRSF1, and CRM1. Created in BioRender. Gaidano, G. (2026) https://BioRender.com/vjnview.

The genomic landscape of RT is shaped by a limited number of recurrent alterations that disrupt fundamental cellular control mechanisms, including cell-cycle regulation, genome integrity, and survival signaling. Among the most consistently affected genes are *TP53*, *CDKN2A*/*B*, *NOTCH1*, and *MYC*, which are altered through deletions, mutations, or chromosomal rearrangements that drive aberrant oncogenic activation (Table 1) [10]. These lesions frequently emerge together within the same transformed clone, indicating functional cooperation in promoting aggressive biological behavior and treatment resistance. Framing RT through these core molecular disruptions provides a conceptual foundation for the detailed discussion of individual pathways that follows (Figure 1).

*TP53* disruption, through mutation and/or 17p deletion, is one of the most frequent genetic events in DLBCL-RT [19]. It can be present in the CLL phase in 10–15% of cases, representing an independent risk factor for RT, or can be acquired at transformation. *TP53* alterations are detected in 60–80% of clonally related DLBCL-type RT and about 20% of clonally unrelated cases [22]. *TP53* encodes p53, a key tumor suppressor that regulates DNA damage response, cell-cycle arrest, DNA repair, and apoptosis [23]. Loss of *TP53* function promotes genomic instability, tumor progression, and resistance to chemotherapy, making *TP53* disruption a central driver of aggressive transformation and treatment refractoriness [24].

Deletion of *CDKN2A* occurs in approximately 30% of DLBCL-type RT and is frequently acquired at the time of transformation, highlighting its role in disease evolution [25]. *CDKN2A* encodes p16^INK4A^ and p14^ARF^, both critical regulators of the G1-S cell-cycle checkpoint and p53 activation. Loss of *CDKN2A* leads to unchecked CDK4/6 activity, enhanced proliferation, and impaired apoptotic signaling [26]. When combined with *TP53* inactivation, *CDKN2A* loss profoundly disrupts cell-cycle control, particularly in the context of hyperactive BCR signaling in CLL, promoting genomic instability and increasing the risk of RT [27].

*NOTCH1* mutations are significantly enriched in DLBCL-type RT (≈30%) compared with CLL at diagnosis (≈8%) [28]. These mutations typically affect the PEST domain, leading to impaired degradation of the active Notch1 intracellular domain and sustained oncogenic signaling [29]. Notch1 activation promotes transcriptional programs involved in proliferation and survival. Clinically, *NOTCH1*-mutated CLL carries a markedly increased cumulative risk of developing RT (up to 45% at 15 years in the CIT era), establishing *NOTCH1* as a major genetic risk factor for transformation, although updated data in the era of targeted therapies are still needed [30].

*c-MYC* alterations contribute to RT pathogenesis by driving proliferation, metabolic reprogramming, self-renewal, and genomic instability [31]. *c-MYC* is a direct transcriptional target of *NOTCH1* signaling and is dysregulated in approximately 40% of DLBCL-RT. Mechanisms include chromosomal translocations involving the IGHV locus, gene amplification, promoter mutations, and loss-of-function mutations in *MGA*, a negative regulator of c-MYC/MAX complexes. These lesions result in sustained c-MYC activation and aggressive tumor behavior [32].

Alterations of the BCR pathway are central to the development of DLBCL-RT. Approximately 50% of clonally related DLBCL-RT display stereotyped immunoglobulin rearrangements, which drive chronic antigen-dependent BCR activation and increased transformation risk [33] (Figure 1). In particular, BCR subset 8 (IGHV4-39/IGHD6-13/IGHJ5) is an independent predictor of RT that promotes leukemic survival through interaction with vimentin in the microenvironment and is frequently associated with trisomy 12 and *NOTCH1* mutations [34]. At the signaling level, *BTK* mutations, also implicated in CLL progression and resistance to BTK inhibitors, are detected in about half of RT cases and sustain oncogenic BCR signaling under therapeutic pressure, while *SF3B1* mutations, often even more frequent, contribute to aberrant RNA splicing, genomic instability, and aggressive disease behavior [33]. Overexpression of ZAP-70 further amplifies IgM-mediated BCR signaling, chemokine production, and protein synthesis, reinforcing proliferative and survival pathways associated with RT [35]. In contrast, NFAT2 functions as a key negative regulator of BCR signaling and disease indolence; its loss, together with disruption of its downstream target LCK, accelerates CLL progression and induces RT-like aggressive disease features in experimental models, highlighting the critical balance between oncogenic BCR activation and anergy-maintaining pathways in RT pathogenesis [36].

### 2.4. Epigenetic Modifications in RT

Epigenetic dysregulation, including aberrant DNA methylation, altered histone marks, and disrupted non-coding RNA networks, plays a central role in RT by reshaping the transcriptional landscape and promoting the aggressive phenotype that distinguishes RT from its CLL precursor (Figure 1).

Genome-wide analyses have shown that clonally related RT preserves much of the CLL epigenetic imprint, but acquires focal hypermethylation at genes involved in stemness (e.g., *OSM*) and cell-cycle control (e.g., *CDKN2A*), driven in part by DNMT dysregulation [37]. *DNMT3A* mutations occur in a minority of RT cases, while *DNMT1* overexpression reinforces silencing of Polycomb-repressed tumor suppressors [37]. In contrast, clonally unrelated RT displays methylation patterns similar to de novo DLBCL, including hypermethylation of *WT1* and immune-regulatory genes [37]. Subtype-specific epigenetic states also influence risk: the IGLV3-21R110 CLL subset shows intermediate methylation and enriched alterations in *SF3B1* and *ATM*, accompanied by epigenetic activation of the oxidative phosphorylation, mTOR, and IRF pathways, which may predispose to transformation [38]. Clonally related RT additionally exhibits global DNA hypomethylation affecting EZH2, Wnt, PI3K/AKT, and IGFR1 signaling, which are associated with therapeutic resistance. Hypermethylation of the *hMLH1* promoter and resulting mismatch-repair defects have been reported in DLBCL-RT, supporting a role for methylation-driven genomic instability [39].

RT is characterized by broad disruption of histone-modifying enzymes and chromatin-remodeling complexes. *EZH2* loss or mutation (≈15% of clonally related RT) reduces H3K27me3-mediated repression and enhances MYC and PI3K/AKT signaling [6,40,41]. Overexpression of *PRMT5*, which symmetrically demethylates histones and non-histone substrates, further promotes oncogenic transcriptional programs [42]. Chromatin remodelers such as *CHD2*, *SETD2*, and *ARID1A* are recurrently mutated, impairing transcriptional fidelity and DNA repair; *CHD2*-deficient RT models demonstrate reduced *PTPRN6* expression, highlighting the functional consequences of remodeling defects [6,40,43].

Furthermore, microRNA (miRNA) deregulation constitutes another key layer of epigenetic disruption. RT displays characteristic miRNA signatures, including downregulation of miR-146b, miR-21, and miR-181b, with upregulation of miR-150 and miR-19b, the latter promoting proliferation by increasing Ki-67 and suppressing p53 [44]. High miR-125a-5p or low miR-34a-5p levels can predict RT with high specificity, and amplification or activation of the miR-17/92 cluster supports *MYC*-driven lymphomagenesis [45]. Extracellular vesicle-derived miRNAs, such as exosomal miR-19b, may further modulate the tumor microenvironment and serve as non-invasive biomarkers of impending transformation [46].

### 2.5. Metabolomic Reprogramming of DLBCL-RT

Metabolic reprogramming has emerged as a defining hallmark of RT, supporting the heightened proliferative and biosynthetic demands of aggressive lymphoma compared with indolent CLL. Multi-omic studies demonstrate that RT cells adopt a distinct OXPHOS^high^-BCR^low^ phenotype marked by increased oxidative phosphorylation, mitochondrial biogenesis, and electron-transport chain activity [6]. This metabolic shift is reinforced by *MYC* activation and *MGA* loss, which amplify OXPHOS-related transcriptional programs, and by enhanced mTORC1 signaling, glycolysis, and ROS-associated pathways that sustain anabolic growth. Complementary transcriptomic and enzymatic analyses further show increased glucose and glutamine uptake, fueling the TCA cycle and lipid and nucleotide synthesis [47]. Inhibition of key regulators such as PI3K or NF-κB suppresses ATP production and glycolysis, while direct targeting of OXPHOS preferentially impairs RT cells, highlighting a lineage-specific metabolic dependency [47]. Genetic lesions common in RT, including alterations in *NOTCH1*, *TP53*, and *CDKN2A*/*B*, further potentiate mitochondrial stress responses and cell-cycle acceleration, integrating metabolic and genomic drivers of transformation [48]. Collectively, these data position metabolic rewiring, particularly heightened mitochondrial OXPHOS and mTOR-driven bioenergetics, as a central feature of RT biology and an emerging therapeutic vulnerability.

**Table 1:** Recurrent copy number variations in Richter transformation.

Genomic Region	Key Gene(s)	CNV	CLL	RT	Note	References
9p21.3	*CDKN2A*/*CDKN2B*	Loss/deletion (often focal; can be biallelic)	7%	30%	Cell-cycle checkpoint	[19,25,40]
17p13 (17p13.3–p11.2)	*TP53*	Loss/deletion (often with additional TP53 disruption)	10%	30–50%	DNA-damage response, apoptosis, genomic stability	[19,25,40]
8q23.3–8q24.3	*MYC*	Gain/amplification (or *MYC* “alterations”)	≤5%	30–35%	Proliferation, metabolism, genomic instability	[19,25,40,49]
13q14.2–q14.3	*DLEU1*/*2*, *MIR15A*/*MIR16-1*	Loss/deletion	50–60%	30–50%	Tumor suppressor microRNAs affecting apoptosis/cell-cycle	[40,50]
11q22–q23	*ATM*	Loss/deletion (CLL lesion); (some RT series also note 11q changes)	10–20%	10–25%	DNA damage response	[19,40]
+12 (whole chr 12)	(multiple; no single driver)	Trisomy 12	10–20%	15–25%	Distinct evolutionary “pathway in RT described in cohort studies	[19,25,40]
2p (often 2p15–2p24 and/or broader 2p gain)	*REL*, *MYCN* (and sometimes broader 2p drivers reported across CLL/RT literature)	Gain/amplification	5–10%	15–30%	NF-κB signaling (REL), proliferation (MYCN)	[25,51]
14q24.1–q32.33	*TRAF3*	Loss/deletion	≤5%	~10–20%	NF-κB pathway regulation (TRAF3 is a negative regulator in B cells)	[40,50]
7q36.2	*EZH2*, *KMT2C*	Deletion	≤5%	~15%	Epigenetic deregulation	[40,52]
6q21–q23	*PRDM1*, *TNFAIP3*	Loss/deletion	5–10%	15–30%	Terminal B-cell differentiation (PRDM1); NF-κB negative regulation (TNFAIP3)	[25,53]
15q15.1	*B2M*	Loss/deletion	0–3%	17–21%	MYC pathway repressorof MHC class I expression)	[19,25,40,54]
9p24.1	*PD-L1*/*PD-L2*	Amplification		~7–8%	Immune escape	[25,50,55,56]

## 3. Tumor Microenvironment of RT

**Figure 2:**
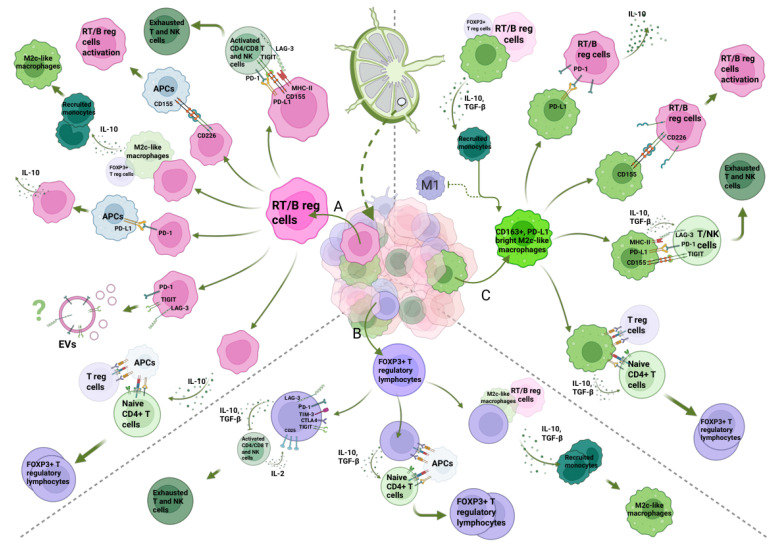
Immune landscape of RT. The RT microenvironment is characterized by a higher density of RT/regulatory B cells, CD163^+^ M2c-like macrophages, and FOXP3^+^ regulatory T cells, as well as a significant increase in immune checkpoint molecules. (**A**) RT/Reg B cells can contribute to the exhaustion of T and NK cells through interactions with inhibitory receptors. These B cells receive activation signals via the CD226/CD155 axis and signals to produce inhibitory cytokines through the PD-1/PD-L1 axis from APCs, and they induce the phenotype of M2c-like macrophages and FOXP3+ regulatory T cells by secreting inhibitory cytokines. In addition, the increased and diverse expression of immune checkpoint molecules on the surface of these cells can be effective in the induction of accelerated exhaustion in newly infiltrating T/NK cells. (**B**) FOXP3^+^ regulatory T cells release inhibitory cytokines such as IL-10, TGF-β, which dampen T and NK-cell activity and promote additional immunosuppressive pathways (affecting M2c-like phenotype in macrophages and expressing FOXP3 in CD4 T cells). (**C**) CD163^+^ M2c-like macrophages may contribute to immune suppression/evasion by producing IL-10 and TGF-β (shifting macrophage polarization away from an M1 phenotype and encouraging Treg differentiation or function), promoting T/NK-cell exhaustion through overexpression of inhibitory ligands (especially PD-L1), interacting with RT/B cells via PD-L1 and CD155. Created in BioRender. Gaidano, G. (2026) https://BioRender.com/vfl49ir.

The tumor microenvironment (TME) in DLBCL-RT comprises immune and non-immune elements that together shape disease biology and treatment response [57] (Figure 2). The immune microenvironment (IME) includes T and B lymphocytes, tumor-associated macrophages (TAMs), myeloid-derived suppressor cells, natural killer (NK) cells, and dendritic cells. In RT, genetic and transcriptional alterations affect not only tumor cell-intrinsic programs (e.g., cell cycle, apoptosis, metabolism) but also B-cell differentiation and T-cell expansion/activation, leading to a reprogrammed IME that promotes immune escape and lymphoma progression [58]. However, many components of the RT TME remain incompletely defined, and current data are largely derived from small cohorts and retrospective analyses. Compared with CLL, RT lymph nodes show broadly similar CD3^+^ and CD8^+^ T-cell infiltration by immunohistochemistry, but are distinguished by increased FOXP3^+^ regulatory T cells and a higher density of CD163^+^ macrophages, together with reduced T-cell receptor clonality in peripheral blood. In addition, the proportion of PD-1^+^ and PD-L1^+^ cells is significantly higher in RT compared to CLL, indicating a more immunosuppressive and functionally exhausted T-cell compartment. Conceptually, the IME of RT fits a PD-L1^+^/TIL^+^ “type I” pattern of adaptive immune resistance, which is generally associated with susceptibility to PD-1/PD-L1 blockade [59].

Immune checkpoint pathways are central to this immunoregulatory network. RT is characterized by upregulation of PD-1/PD-L1, CTLA-4, LAG-3, TIM-3, and TIGIT on tumor and immune cells [60]. A distinctive feature of DLBCL-RT is the frequent expression of PD-1 on neoplastic B cells, in contrast to DLBCL [21,59]. In immunohistochemical series, PD-1 positivity on tumor B cells was observed in most DLBCL-RT samples but rarely in other DLBCL, and strongly correlated with clonal relatedness to CLL. PD-L1, by contrast, is usually expressed on bystander histiocytes and dendritic cells rather than on RT B cells [21,59]. This pattern supports the concept that PD-1^high^ malignant B cells may adopt regulatory B-cell–like properties: upon engagement of PD-1 with PD-L1 on myeloid cells, these cells can produce immunosuppressive cytokines such as IL-10 and TGF-β, thereby dampening antitumor T- and NK-cell activity and promoting expansion of FOXP3^+^ Tregs and M2-like macrophages [61]. These observations are consistent with broader cancer immunology data showing that PD-1- or PD-L1-expressing regulatory B cells suppress effector T cells via IL-10 and PD-1/PD-L1 signaling in solid tumors, such as hepatocellular carcinoma and differentiated thyroid cancer [58]. In RT, emerging evidence from translational studies suggests that a subset of malignant B cells co-expresses PD-1 and its ligands (PD-L1/PD-L2) and secretes IL-10 and TGF-β, reinforcing a self-sustaining immunosuppressive circuit [58]. Notably, the same PD-1–SHP2 inhibitory axis can have distinct functional consequences depending on whether it is engaged on T cells (driving exhaustion) or on B cells (inducing an immunoregulatory phenotype) [62] (Figure 2).

LAG-3 is another key checkpoint molecule in RT. It is expressed on activated CD4^+^ and CD8^+^ T cells and NK cells, and can also be detected on malignant B cells in a subset of RT samples [61,63]. LAG-3 primarily binds MHC class II with high affinity, but additional ligands such as galectin-3, LSECtin, and FGL1 broaden its immunomodulatory role [64]. In RT, LAG-3 is commonly expressed on both tumor cells and tumor-infiltrating lymphocytes and correlates with higher HLA class I and II expression, indicating that antigen presentation pathways remain intact, but downstream T-cell responses are attenuated [63]. Engagement of LAG-3 on T and NK cells by MHC class II on antigen-presenting cells or RT cells delivers inhibitory signals that blunt CD4^+^ helper and CD8^+^ cytotoxic responses, thereby facilitating immune escape [64].

The TIGIT-CD226 axis provides an additional checkpoint layer in DLBCL-RT. TIGIT is expressed on activated CD4^+^ and CD8^+^ T cells, γδ T cells, Tregs, and NK cells, and binds several ligands on antigen-presenting and tumor cells, including CD155 (its highest-affinity partner) and CD112 [65]. Binding of TIGIT to CD155 delivers inhibitory signals and competes with the costimulatory receptor CD226 (DNAM-1), which engages the same ligands but with lower affinity, shifting the balance toward T- and NK-cell exhaustion. In RT, TIGIT expression on neoplastic B-cells appears aberrantly increased, while CD226 is also upregulated. This suggests that CD226-CD155 interactions on RT cells may enhance BCR signaling and promote activation, whereas TIGIT skewing on T/NK cells favors an immunosuppressive milieu [66]. Thus, the TIGIT-CD226 axis in DLBCL-RT simultaneously influences tumor cell activation and effector cell exhaustion [66].

FOXP3^+^ regulatory T cells in RT are increased in nodal and tissue microenvironments, but their functional impact is more nuanced than in solid tumors [59]. In many solid cancers, Treg accumulation is associated with poor prognosis, whereas in lymphoid malignancies their role is context-dependent [67]. In CLL, higher Treg frequencies correlate with adverse outcomes, whereas in RT, FOXP3^+^ Tregs may serve as predictive biomarkers of response to PD-1 blockade, with higher baseline infiltration observed in responders to pembrolizumab in early studies [68,69]. Mechanistically, Tregs in RT are likely to exert classical suppressive functions, including the secretion of IL-10, TGF-β, and IL-35; high expression of CTLA-4, PD-1, LAG-3, and TIGIT; metabolic competition for IL-2; adenosine production; and direct cytotoxicity against activated immune cells, although the relative contribution of each mechanism in RT remains to be fully defined [70].

TAMs represent another major immunoregulatory population in RT [57,71]. Most TAMs derive from circulating monocytes recruited into the TME and are skewed toward an M2-like phenotype. While the M1/M2 dichotomy oversimplifies macrophage biology, M2c-like macrophages, characterized by high CD163, MerTK, and Tie2 expression, are particularly linked to immune suppression and tissue remodeling [72]. RT lesions exhibit increased infiltration of CD163^+^ M2-skewed TAMs compared with CLL, and these cells frequently express high levels of PD-L1 [59,73]. Although detailed transcriptional subtyping of TAMs in DLBCL-RT is lacking, it is plausible that they share features with IL-10–polarized M2c macrophages. Potential roles include: (i) producing IL-10 and TGF-β, thereby shifting macrophage polarization away from M1, suppressing T/NK responses, and promoting Treg differentiation; (ii) engaging PD-1, TIGIT, and LAG-3 on T and NK cells via PD-L1, CD155, and MHC class II, driving exhaustion; and (iii) interacting directly with RT B cells through PD-L1–PD-1 and CD155-CD226 axes to induce IL-10 production and enhance B-cell activation [74].

Taken together, DLBCL-RT is embedded in a profoundly immunosuppressive microenvironment characterized by PD-1^high^ regulatory-like malignant B cells, exhausted and clonally restricted T cells, expanded FOXP3^+^ Tregs, and CD163^+^ M2-skewed TAMs, all interconnected by redundant checkpoint and cytokine networks. This complex IME not only fuels disease progression and therapeutic resistance but also provides a strong biological rationale for combined strategies that target both tumor-intrinsic pathways and immune escape mechanisms in RT [75].

## 4. Targeted Treatment for DLBCL-RT

The therapeutic landscape for DLBCL-RT largely relies on CIT regimens used for de novo aggressive DLBCL, such as R-CHOP (rituximab, cyclophosphamide, doxorubicin, vincristine, and prednisone), which remains a standard first-line option but still yields unsatisfactory clinical outcomes [76]. Given the highly aggressive nature of DLBCL-RT, the primary aim of initial therapy is typically to reduce tumor burden sufficiently to allow eligible patients to proceed to allogeneic hematopoietic stem cell transplantation (allo-HSCT) [77]. Nevertheless, complete remission (CR) rates with R-CHOP generally remain close to 20%, and reported median OS spans only 6–12 months [78].

A retrospective evaluation of R-EPOCH (rituximab, etoposide, prednisone, vincristine, cyclophosphamide, and doxorubicin) as first-line therapy showed a median OS of merely 5.9 months and highlighted markedly poorer outcomes in patients with *TP53* deletions or complex karyotype [12]. Hyper-CVXD (fractionated cyclophosphamide, vincristine, liposomal daunorubicin, and dexamethasone) was studied in a phase II trial and, although it produced a clinically meaningful CR rate of 38%, it was accompanied by substantial toxicity, with an overall mortality of 20% [79]. An additional phase I/II investigation of the OFAR2 regimen (oxaliplatin, fludarabine, ara-C, and rituximab) demonstrated an overall response rate (ORR) of 38.7%, including a 6.5% CR rate and a median survival of just 6.6 months [80]. The CHOP-OR phase II trial assessed CHOP-O (cyclophosphamide, doxorubicin, vincristine, and prednisolone, combined with induction and maintenance therapy using the anti-CD20 monoclonal antibody ofatumumab) in newly diagnosed DLBCL-RT [81]. The study reported an ORR of 46% and a CR rate of 27%, while the median progression-free survival (PFS) of 6.2 months and median OS of 11.4 months did not demonstrate improvement over outcomes typically achieved with R-CHOP. Collectively, these results emphasize the inadequacy of standard CIT and the urgent need for alternative therapeutic approaches.

In recent years, emerging targeted therapy strategies, namely pathway inhibitors, immune checkpoint inhibitors, T-cell–engaging therapies, and ADCs, have shown promising outcomes and may help redefine the treatment paradigm for DLBCL-RT [82]. A summary of currently ongoing clinical trials with targeted therapeutic agents in this setting is presented in Table 2.

### 4.1. Pathway Inhibitors in DLBCL-RT

**Figure 3:**
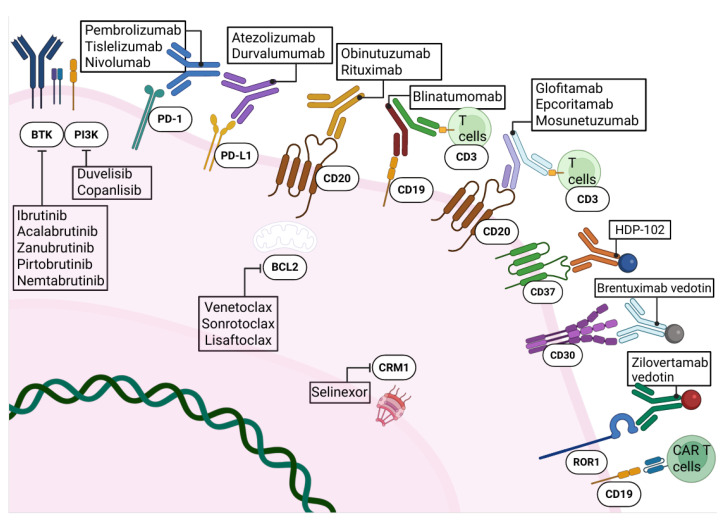
Current and emerging therapeutic agents of RT. Therapeutic strategies include anti-CD20 mAbs; immune checkpoint blockade (PD-1/PD-L1), often combined with targeted agents; inhibition of BCR-pathway signaling with BTKi or PI3Ki; inhibition of BCL2-mediated antiapoptotic signaling (BCL2i); inhibition of CRM1-dependent nuclear export; T-cell–engaging bispecific antibodies (CD19 × CD3 or CD20 × CD3); ADCs targeting CD30, ROR1, or CD37; and anti-CD19 CAR-T cell therapy. Abbreviations: mAb, monoclonal antibody; BTKi, BTK inhibitor; PI3Ki, PI3K inhibitor; BCL2i, BCL2 inhibitor; ADC, antibody-drug conjugate; CAR, chimeric antigen receptor; ROR1, receptor tyrosine kinase-like orphan receptor 1. Created in BioRender. Gaidano, G. (2026) https://BioRender.com/5phlu9z.

Covalent BTK inhibitors (BTKi) act by targeting the Cys481 residue of BTK, located within the ATP-binding pocket of the enzyme, thereby disrupting the downstream BCR signaling [83] (Figure 3). By occupying this ATP-binding site, BTKi agents prevent the phosphorylation of downstream signaling molecules such as Akt and PLCγ2, ultimately inhibiting key steps of the BCR cascade [84,85]. As a result, BTK-mediated signaling is effectively blocked, a mechanism confirmed through both in vitro and in vivo studies [86].

Ibrutinib, the first covalent BTKi to be approved for CLL and mantle cell lymphoma treatment, forms an irreversible bond with Cys481, but in DLBCL-RT it has demonstrated only modest efficacy, with consistently disappointing clinical outcomes across multiple analyses [87,88,89,90]. Comparable results were observed with acalabrutinib, a second-generation covalent BTKi examined in the ACE-CL-001 study, where patients achieved a CR rate of 8% and a median PFS of just 3 months [91]. Better results emerged from a phase II study (NCT02343120) evaluating the second-generation covalent BTKi zanubrutinib, which reported a CR rate of 15.4%, although the trial was limited by its small sample size (*n* = 13) [92].

Resistance to covalent BTKi is often driven by prior BTKi exposure during the antecedent CLL phase, resulting in acquired BTK mutations (particularly those affecting Cys481) and gain-of-function alterations in *PLCG2* [84,85,93]. In order to overcome covalent BTKi refractoriness, pirtobrutinib, a reversible third-generation non-covalent BTKi, was developed [85]. Pirtobrutinib binds BTK through an allosteric blocking mechanism involving hydrogen bonding and pi-stacking interactions near the active site without forming a covalent bond to Cys481, thus retaining activity against most BTKi-resistant BTK variants [94]. Consequently, pirtobrutinib has demonstrated robust efficacy in CLL previously treated with covalent BTK inhibitors [84]. In the DLBCL-RT cohort of the phase 1/2 BRUIN study (NCT03740529), pirtobrutinib yielded an ORR of 50% and a CR rate of 13% in patients who were frequently pre-exposed to covalent BTKi; however, the median PFS remained limited at 3.7 months [95]. A recent phase II trial (NCT05536349) evaluating the combination of pirtobrutinib with the anti-CD20 monoclonal antibody (mAb) obinutuzumab and the BCL-2 inhibitor venetoclax demonstrated highly encouraging activity in DLBCL-RT, with an ORR of 67% [96]. At 12 months, event-free survival (EFS) and OS for the entire cohort were 73% and 82%, respectively. Remarkably, among patients with relapsed/refractory (R/R) RT, 12-month EFS and OS rates were 75% and 88%, respectively. Moreover, an emerging reversible non-covalent BTKi, nemtabrutinib (MK-1026), has shown superior antitumor activity compared to ibrutinib in CLL and aggressive B-cell lymphoma murine models [97]. Two clinical studies involving DLBCL-RT patients (NCT03162536 and NCT04728893) are currently ongoing.

BCL-2 inhibition represents another promising pathway-targeting strategy in DLBCL-RT [93] (Figure 3). Venetoclax, which displayed great efficacy in CLL management, represents a current therapeutic strategy in both treatment-naïve and R/R CLL [98,99,100]. In a phase I study (NCT01328626), venetoclax monotherapy produced an ORR of 43% in patients with DLBCL-RT, although no CR were observed [101]. More encouraging findings came from a phase II trial (NCT03054896) investigating venetoclax in combination with R-DA-EPOCH, which achieved an ORR of 62% and an impressive CR rate of 50% [102]. Furthermore, a phase II trial evaluating venetoclax with R-CHOP enrolled 27 DLBCL-RT patients (NCT03054896), reporting an ORR of 68% and a CR rate of 48%, with a median PFS of 7.2 months and median OS of 19.5 months [103]. Newer BCL-2 inhibitors, including lisaftoclax and sonrotoclax, deserve to be also tested in the context of RT [104,105].

### 4.2. Immune Checkpoint Inhibitors in DLBCL-RT

As stated above, the PD-1/PD-L1 interaction drives immune suppression through both intercellular and extracellular signals, leading to cancer immune evasion [106] (Figure 3). Targeting the PD-1 axis with mAbs that block the PD-1/PD-L1 binding has become a validated strategy across both solid tumors and hematologic malignancies [107]. Pembrolizumab, an anti-PD-1 mAb, was examined in a phase II trial (NCT02332980), where DLBCL-RT patients achieved an ORR of 40% and a median OS of 11 months [102]. Importantly, this cohort was heterogeneous, and 70% of participants had received prior ibrutinib therapy, which may have influenced outcomes [108].

Pembrolizumab monotherapy was further evaluated in the KEYNOTE-170 phase II study, including 21 patients with R/R RT; however, results were poor for those with R/R DLBCL-RT, with an ORR of 4.8% and no CR [109]. Given the preclinical and clinical evidence suggesting synergy between BTKi agents and immune checkpoint blockade, co-targeting PD-1 and pathways associated with BCR signaling may enhance therapeutic effectiveness [110]. In particular, the combination of pembrolizumab with BCR signaling inhibitors, namely the BTKi ibrutinib and the PI3K inhibitor (PI3Ki) idelalisib, was associated with improved treatment responses, achieving an ORR of 62.5%, with a CR rate of 25% CR and partial response (PR) rate of 37.5% [111].

The anti-PD-1 mAb tislelizumab has been evaluated in combination with zanubrutinib, with or without the BCL-2 inhibitor sonrotoclax, in the phase 2 RT1 trial [112]. Among the 48 patients included in the full efficacy analysis, the ORR reached 58.3%, including a CR rate of 39.6%. Median OS was not reached, and the two-year OS rate was 63.3%. In addition, nivolumab, an anti-PD-1 mAb, has been tested with ibrutinib in a phase 2 trial (NCT02420912) that included 24 treatment-naïve and R/R DLBCL-RT patients [113]. The combination achieved an ORR of 41.7%, a CR rate of 33.3%, and a median OS of 25 months, with superior outcomes in first-line patients. Furthermore, the MOLTO trial (NCT04082897) assessed a triplet regimen of the PD-L1 inhibitor atezolizumab, obinutuzumab, and venetoclax, demonstrating promising efficacy in DLBCL-RT [114]. Among patients evaluable for clonality (*n* = 24), 83% had clonally related disease. ORR was 67.9%, and CR was 28.6%, while 12-month PFS and OS were 42.9% and 64.3%, respectively. Collectively, these data indicate that, while PD-1/PD-L1 blockade alone has limited and inconsistent efficacy in RT, this pathway remains a key therapeutic target when incorporated into rational combination regimens that also address BCR signaling, apoptosis pathways, and the immunosuppressive microenvironment. This supports a model in which checkpoint inhibition helps restore T-cell function, but requires concurrent tumor-intrinsic targeting to achieve durable disease control.

### 4.3. T-Cell Engaging Therapies

Harnessing T-cell immunity offers another powerful therapeutic strategy. In de novo DLBCL, both bsAbs and CAR-T cell therapies have demonstrated substantial activity and are now included in treatment algorithms [115,116] (Figure 3). BsAbs consist of engineered molecules capable of binding two distinct epitopes or antigens simultaneously [106]. BsAbs mode of action centers on recruiting T cells into proximity with malignant B cells, thereby enabling direct cytotoxicity independent of endogenous antigen specificity [117,118].

Promising early findings were reported from a monocentric phase II trial (NCT03121534) studying blinatumomab, a CD19 × CD3 bsAb, in nine RT patients, four of whom experienced reductions in nodal disease, including one CR lasting more than one year [119]. Blinatumomab has also been investigated in combination with CIT in the BLINART phase II study, which evaluated blinatumomab as an induction therapy for DLBCL-RT following R-CHOP debulking [120]. In the subgroup of patients not achieving CR with R-CHOP, blinatumomab provided an ORR of 36% and a CR rate of 20%, with manageable toxicity.

Glofitamab, a CD3 × CD20 bsAb, was examined in a phase 1/2 trial involving 11 heavily pretreated and CD20-refractory R/R RT patients, yielding noteworthy initial responses [121]. Even more encouraging results have come from the phase I/II trial EPCORE CLL-1 (NCT04623541), assessing the CD20 × CD3 bsAb epcoritamab in DLBCL-RT [122]. Nearly half of the cohort had prior CIT and 49% had *TP53* mutations; overall, the ORR was 47.6%. Notably, first-line patients demonstrated higher response rates, with an ORR of 57.1% and a CR rate of 52%.

CD19-directed autologous CAR-T therapy has transformed the management of R/R B-cell malignancies, offering high response rates and durable remissions [116]. CAR-T cell products incorporate an extracellular CD19-targeting domain coupled to an intracellular co-stimulatory domain (e.g., 4-1BB or CD28) and a CD3ζ signaling domain that promotes T-cell activation, expansion, and persistence [123,124]. The workflow of CAR-T therapy involves leukapheresis, ex vivo genetic engineering and expansion of CAR-T cells, lymphodepleting chemotherapy, and subsequent infusion [125]. A multicenter retrospective analysis of 69 DLBCL-RT patients treated with CD19-directed CAR-T reported an ORR of 63.8% and a CR rate of 46.3%; patients who attained CR had a median duration of response of 27.6 months. After a median follow-up of 24 months, median PFS and OS were 4.7 and 8.5 months, respectively [126]. Moreover, a larger registry-based study using Center for International Blood and Marrow Transplant Research (CIBMTR) data also supported the activity of CD19-CAR-T in DLBCL-RT, reporting an ORR of 71% and a CR rate of 57% among 128 evaluable patients. At a median follow-up of 25 months, 2-year PFS and OS were 32% and 46%, with relapse/progression and treatment-related mortality rates of 59% and 9%, respectively [127]. Zamtocabtagene autoleucel, a bispecific CD3 × CD19 × CD20 CAR-T product, is currently under investigation in the phase 2 DALY II USA study for R/R DLBCL, which includes a DLBCL-RT cohort [128]. Initial analyses showed an ORR of 72.9% and a CR rate of 49.2%, with 12-month PFS and OS rates of 42% and 72% in DLBCL. However, results specifically for the RT subgroup have not yet been disclosed.

Given the immune dysfunction in CLL discussed above, CAR-T cells derived from patients with DLBCL-RT (i.e., autologous CAR-T cells) may be intrinsically dysfunctional compared with those from patients with de novo DLBCL, potentially resulting in reduced efficacy [106,129]. Accordingly, an emerging and promising therapeutic strategy is the use of off-the-shelf allogeneic CAR-T cells, which have demonstrated encouraging efficacy in early phase I trials in R/R large B-cell lymphoma [130]. Unlike autologous CAR-T products, allogeneic CAR-T cells are generated from healthy donors with intact T-cell immunity. Key remaining challenges include host rejection and graft-versus-host disease (GVHD), which may be mitigated through HLA matching, inhibition of T-cell receptor (TCR) signaling, such as by using virus-specific T cells (VSTs), memory T cells, or γδ T cells, or by TCR gene disruption via gene-editing technologies [129].

### 4.4. Antibody-Drug Conjugates (ADCs)

ADCs represent an emerging and highly targeted therapeutic approach for RT. ADCs consist of a mAb linked chemically to a cytotoxic payload, enabling selective delivery of potent agents to malignant cells while minimizing systemic exposure [131] (Figure 3). Following binding to a specific surface antigen on tumor cells, ADCs are internalized, and the linker is cleaved intracellularly, releasing the cytotoxic compound and inducing cell death. DLBCL-RT has been explored using recently developed murine patient-derived xenograft (PDX) models, which facilitate the investigation of tumor biology and preclinical therapeutic responses despite the rarity of human cases [132,133]. Zilovertamab vedotin, an ADC carrying monomethyl auristatin E and directed against ROR1, has shown substantial activity in preclinical studies utilizing PDX models [134]. ROR1 is an embryonic tyrosine kinase receptor that regulates cell survival, proliferation, and migration through non-canonical WNT signaling [135]. It is largely absent in normal adult tissues, including mature B cells, but is overexpressed in both CLL and DLBCL-RT [136,137]. In PDX models, zilovertamab vedotin markedly reduced tumor burden and prolonged survival, with no treatment-related toxicities observed [134]. A phase 1 clinical trial evaluating this agent in relapsed/refractory lymphomas included one patient with DLBCL-RT, and a dedicated phase II trial (NCT05458297) was subsequently initiated [138].

## 5. Conclusions

In conclusion, DLBCL-type RT exemplifies the convergence of early evolutionary priming, genome/epigenome instability, and microenvironmental immune paralysis into a clinically explosive lymphoma state. The emerging picture is not of a sudden stochastic “switch”, but of transformation-prone lineages that can persist for years beneath the detection threshold, accumulate cooperating lesions (TP53–CDKN2A/B–NOTCH1–MYC and others), and ultimately exploit a permissive CLL niche to expand under therapeutic pressure. This framework has two immediate implications. First, RT should be approached as a disease of dynamic clonal ecosystems, where targeted agents reshape selection more than they eradicate risk; thus, reassessing RT biology in the post-BTKi/BCL2i era is essential. Second, the immune contexture described here—PD-1^high^ malignant B cells, PD-L1^+^ myeloid networks, exhausted and clonally restricted T cells, and enriched Tregs and M2-skewed macrophages—suggests that “immune presence” is insufficient: what matters is immune architecture and checkpoint circuitry. The treatment landscape of DLBCL-RT remains largely dominated by CIT; however, several targeted therapeutic strategies are emerging, particularly BTKi, immune checkpoint inhibitors, and bsAbs. While single-agent covalent BTKi and immune checkpoint inhibitors have demonstrated disappointing outcomes in DLBCL-RT, early signals from combinatorial strategies (BTK/BCL2 inhibition with PD-1 blockade; bsAbs; CAR-T) indicate that durable control is achievable, but only if therapies are matched to the dominant resistance layer, i.e., tumor-intrinsic escape, immune exclusion/dysfunction, or both. In addition, promising activity has been reported with the non-covalent BTKi pirtobrutinib and with the CD3 × CD20 bsAb epcoritamab in DLBCL-RT. These findings have paved the way for the initiation of phase III clinical trials, including CLL-RT2 and PIRAMID, in the first-line setting for DLBCL-RT. Both trials compare standard-of-care CIT with pirtobrutinib plus epcoritamab in CLL-RT2 and pirtobrutinib combined with R-CHOP in PIRAMID in treatment-naïve DLBCL-RT, and are expected to begin enrollment soon. Future progress will depend on integrating ultra-sensitive detection of high-risk subclones with spatial and systemic immune profiling to enable earlier interception, rational combinations, and biomarker-driven trials tailored to the unique biology of RT.

## Figures and Tables

**Table 2:** Ongoing clinical trials with target therapy in DLBCL-RT (last accessed on 11 December 2025).

NCT Code	Phase	Intervention	Target	Primary Endpoint
NCT06926205	II	CHOP + Mosunetuzumab	CD20	CR rate
NCT06186648	II	R/G-CHOP + Glofitamab	CD20	CR rate
NCT04271956	II	Zanubrutinib + Tislelizumab +/− Sonrotoclax	BTK, PD-1, BCL2	ORR
NCT03054896	II	R-CHOP/DA-EPOCH-R + Venetoclax	CD20, BCL2	CR rate
NCT03899337	II	R-CHOP +/− Acalabrutinib	CD20, BTK	PFS, ORR
NCT06676033	I	Epcoritamab	CD20	Immunophenotype and transcriptome of T-cells and tumor cells in lymph node and bone marrow
NCT04679012	II	R-EPOCH + Polatuzumab vedotin	CD20, CD79b	CMR/CR rate
NCT03534323	I/II	Duvelisib + Venetoclax	PI3K, BCL2	MTD, CR rate
NCT04082897	II	Obinutuzumab + Atezolizumab + Venetoclax	CD20, PD-L1, BCL2	ORR
NCT05388006	II	Acalabrutinib + Venetoclax + Durvalumab	BTK, BCL2, PD-L1	PFS
NCT06863402	II	Nemtabrutinib + Pembrolizumab	BTK, PD-1	ORR, quality of life
NCT02846623	II	Atezolizumab + Obinutuzumab + Venetoclax	PD-L1, CD20, BCL2	MRD negative rate
NCT07220187	III	R-CHOP +/− Pirtobrutinib	CD20, BTK	PFS, OS
NCT05458297	II	Zilovertamab vedotin	ROR-1	ORR
NCT05536349	II	Pirtobrutinib + Venetoclax + Obinutuzumab	BTK, BCL2, CD20	ORR
NCT05672173	II	Lisocabtagene maraleucel + Nivolumab + Ibrutinib	CD19, PD-1, BTK	CR rate, UT rate
NCT07101328	I	LY4152199	BAFF-R	DLT rate, ORR
NCT03884998	I	Copanlisib + Nivolumab	PI3K, PD-1	DLT rate, AE rate
NCT06735664	I	Zanubrutinib + Odronextamab	BTK, CD20	DLT rate, AE rate
NCT03321643	I	R-GEMOX + Atezolizumab	CD20, PD-L1	AE rate, MTD
NCT07168486	I	LTG2950	CD19, CD20, CD22	AE rate, ORR, CAR-T cell persistence and functionality
NCT04792489	II	Zamtocabtagene autoleucel	CD19, CD20	ORR
NCT04771572	I	LP-118	BCL2, BCL-XL	PK profile, MTD
NCT05828589	I	BGB-21447	BCL2	DLT rate, AE rate, TLS rate
NCT05107674	I	NX-1607	CBL-B	AE rate, ORR
NCT03479268	I	Pevonedistat + Ibrutinib	NAE, BTK	DLT rate, AE rate
NCT06561425	I/II	GLPG5101	CD19	DLT rate, AE rate, ORR
NCT05025800	I/II	R^2^ + ALX148	CD20, CRBN, CD47	Recommended phase II dose, CR rate

Abbreviations: *CHOP*, cyclophosphamide-doxorubicin-vincristine-prednisone; *CR*, complete response; *R-CHOP*, rituximab-CHOP; *G-CHOP*, obinutuzumab-CHOP; *ORR*, overall response rate; *BTK*, Bruton tyrosine kinase; *PD-1*, programmed death-1; *BCL2*, B-cell lymphoma 2; *PFS*, progression-free survival; *DA-EPOCH-R*, dose adjusted etoposide-prednisone-vincristine-cyclophosphamide-doxorubicin-rituximab; *CMR*, complete metabolic response; *PI3K*, phosphoinositide 3-kinase; *MTD*, maximum tolerated dose; *PD-L1*, programmed death-ligand 1; *MRD*, minimal residual disease; *OS*, overall survival; *ROR-1*, receptor tyrosine kinase-like orphan receptor 1; *UT*, unacceptable toxicity; *BAFF-R*, B-cell activating factor receptor; *DLT*, dose-limiting toxicity; *AE*, adverse event; *CAR*, chimeric antigen receptor; *BCL-XL*, B-cell lymphoma-extra large; *PK*, pharmacokinetics; *TLS*, tumor lysis syndrome; *CBL-B*, casitas B lymphoma-B; *R-GEMOX*, rituximab-gemcitabine-oxaliplatin; *NAE*, NEDD8 activating enzyme.

## Data Availability

No new data were created or analyzed in this study.
